# Food and Food Products on the Italian Market for Ketogenic Dietary Treatment of Neurological Diseases

**DOI:** 10.3390/nu11051104

**Published:** 2019-05-17

**Authors:** Alessandro Leone, Ramona De Amicis, Chiara Lessa, Anna Tagliabue, Claudia Trentani, Cinzia Ferraris, Alberto Battezzati, Pierangelo Veggiotti, Andrea Foppiani, Simone Ravella, Simona Bertoli

**Affiliations:** 1 International Center for the Assessment of Nutritional Status (ICANS), Department of Food Environmental and Nutritional Sciences (DeFENS), University of Milan, Via Sandro Botticelli 21, 20133 Milan, Italy; ramona.deamicis@unimi.it (R.D.A.); chiara.lessa@unimi.it (C.L.); alberto.battezzati@unimi.it (A.B.); andrea.foppiani@unimi.it (A.F.); ravella.simone@gmail.com (S.R.); simona.bertoli@unimi.it (S.B.); 2 Human Nutrition and Eating Disorder Research Center, Department of Public Health, Experimental and Forensic Medicine, University of Pavia, Via Agostino Bassi 21, 27100 Pavia, Italy; anna.tagliabue@unipv.it (A.T.); claudia.trentani@unipv.it (C.T.); cinzia.ferraris@unipv.it (C.F.); 3 Pediatric Neurology Unit, “V. Buzzi” Hospital, Via Lodovico Castelvetro 32, 20154 Milan, Italy; pierangelo.veggiotti@unimi.it

**Keywords:** ketogenic diet, food, epilepsy, glucose transporter 1 deficiency syndrome, pyruvate dehydrogenase deficiency, children, adult

## Abstract

The ketogenic diet (KD) is the first line intervention for glucose transporter 1 deficiency syndrome and pyruvate dehydrogenase deficiency, and is recommended for refractory epilepsy. It is a normo-caloric, high-fat, adequate-protein, and low-carbohydrate diet aimed at switching the brain metabolism from glucose dependence to the utilization of ketone bodies. Several variants of KD are currently available. Depending on the variant, KDs require the almost total exclusion, or a limited consumption of carbohydrates. Thus, there is total avoidance, or a limited consumption of cereal-based foods, and a reduction in fruit and vegetable intake. KDs, especially the more restrictive variants, are characterized by low variability, palatability, and tolerability, as well as by side-effects, like gastrointestinal disorders, nephrolithiasis, growth retardation, hyperlipidemia, and mineral and vitamin deficiency. In recent years, in an effort to improve the quality of life of patients on KDs, food companies have started to develop, and commercialize, several food products specific for such patients. This review summarizes the foods themselves, including sweeteners, and food products currently available for the ketogenic dietary treatment of neurological diseases. It describes the nutritional characteristics and gives indications for the use of the different products, taking into account their metabolic and health effects.

## 1. Introduction

The ketogenic diet (KD) is a normo-caloric, high-fat, adequate-protein, and low-carbohydrate dietary treatment that, according to recent international guidelines, is recommended for children and adults with drug-refractory epilepsy and two rare neurometabolic disorders: glucose transporter 1 deficiency syndrome (GLUT1-DS) and pyruvate dehydrogenase (PDH) deficiency [1,2,3]. The main goal of KD is to mimic the metabolic condition that occurs during starvation. Indeed, in conditions of restricted carbohydrate and high-fat availability, the brain metabolism shifts from glucose dependence to the utilization of ketone bodies, principally β-hydroxybutyrate (BHB) and acetoacetate (ACA), produced in the liver by the oxidation of fatty acids [4].

Despite KD having been used for the first time in 1921 to treat patients with drug-refractory epilepsy, the mechanisms by which KD exerts its anti-seizure effects are not completely understood. Multiple hypotheses have been put forward to explain the KD effects. One hypothesis involves the modulation of brain neurotransmitters, including γ-aminobutyric acid (GABA), glutamate, and adenosine, as well as ion channel regulation [5,6,7]. It has been proposed that ketone bodies alter the metabolism of glutamate in such a way that the levels of GABA increase and inhibitory neurotransmission is enhanced [8]. BHB and ACA have also been shown to influence presynaptic glutamate release by directly competing with Cl− for the allosteric activation of vesicular glutamate transporters, resulting in diminished glutamate release [9]. Ketone bodies, specifically BHB and ACA, can also open ATP-sensitive potassium channels, potentially reducing the release of neurotransmitters, and the neuron’s action potential [10,11]. Moreover, the increased ATP synthesis leads to the accumulation of adenosine, stimulating adenosine A1 receptors and reducing spontaneous seizures [12,13].

KD can also exert a neuroprotective effect. It is known that the epileptogenic state involves complex molecular pathways in which oxidative stress and mitochondrial dysfunction can play important roles in neuronal cell death [14]. However, KD has been shown to enhance energy reserves, ATP levels, and the expression of many enzymes involved in multiple metabolic pathways in the mitochondria, and it increases mitochondrial biogenesis in the hippocampus [5,15]. Additionally, many KD studies have demonstrated an elevated antioxidant activity, diminished reactive oxygen species’ (ROS) production, and decreased ROS-induced damage [4,5,16,17], due to NADH oxidation, upregulation of the mitochondrial uncoupling protein (UCP) activity, and the biosynthesis of glutathione [18].

Finally, recent findings have led to the speculation of an important role for neuroactive peptides, such as insulin, leptin, ghrelin, des-acyl ghrelin, and adiponectin, in regulating neuronal activity during seizures [19]. Further details on KD mechanisms can be found in numerous reviews on this subject [4,5,6,7].

The mechanism of action of KD in the treatment of neurometabolic disorders is clearer: in the brain, the ketone bodies act as an alternative fuel to glucose. GLUT1-DS is caused by a defect in the protein responsible for the transfer of glucose across the blood-brain barrier, and is manifested in seizures early in life and impairment of brain growth with developmental delay and other neurological problems, including a complex movement disorder. The use of ketone bodies as an alternative fuel to glucose bypasses the deficit of the glucose transporter, as the ketone bodies can enter the brain via facilitated diffusion mediated by the MCT1 transporter [3]. Instead, PDH is caused by an enzyme deficiency within the pyruvate dehydrogenase complex, which allows the metabolism of pyruvate to acetyl-coA, which, in turn, enters the Krebs cycle [20]. Thus, in PDH patients, because of this enzyme deficit, the pyruvate is metabolized in lactate, resulting in a compromised ATP production. KD allows this metabolic block to be bypassed [21].

In addition to the above described effects, there is emerging evidence that ketone bodies exhibit systemic and neuroprotective anti-inflammatory effects [22], as KD treatment reduces the expression of pro-inflammatory cytokines, microglial activation, pain, and inflammation [23,24,25]. This occurs through the fatty acid-induced activation of the peroxisome proliferator-activated receptor α (PPARs), which, in turn, inhibits the pro-inflammatory transcription factor nuclear factor kappa B pathway, and the ability of BHB to bind the hydroxycarboxylic acid receptor 2 and inhibit the innate immune sensor NOD-like receptor 3 inflammasome that controls the activation of caspase-1 and the release of the pro-inflammatory cytokines interleukin (IL)-1β and IL-18 [25]. 

Thanks to investigations that have revealed the favorable impact of ketone bodies on cellular metabolism in many tissues, KD has also been evaluated for numerous other neurological and neuromuscular disorders [26,27], like mitochondrial, Parkinson’s, and Alzheimer’s disease, migraine/headache, depression, glycogenosis, high grade glioblastoma [28], and amyotrophic lateral sclerosis, as well as for metabolic and endocrine disorders like diabetes, obesity, metabolic syndrome, poly-cystic ovary syndrome, and congenital hyperinsulinism [29,30,31]. Recent reviews have summarized the studies exploring the effects of KDs in the treatment of specific neurological disorders, and the clinical studies investigating the efficacy of KDs in the management of different neurological disorders in adults [22,27].

**Figure 1:**
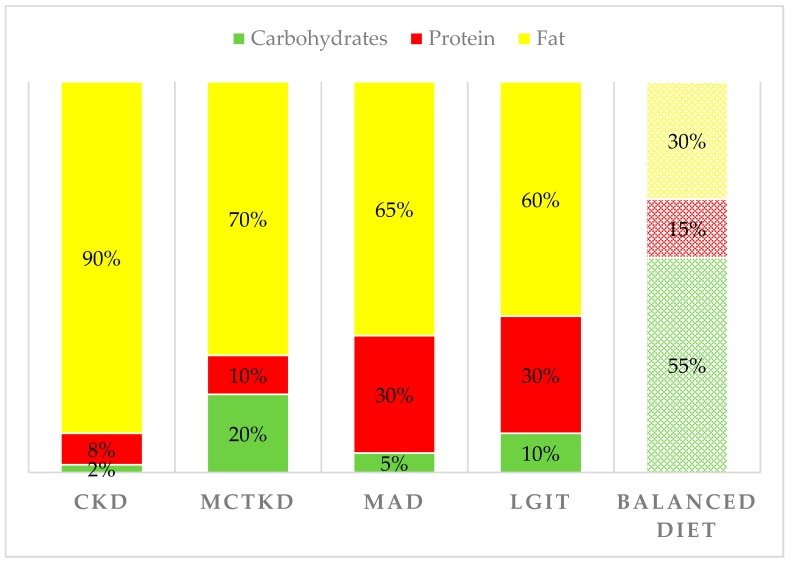
Proportion of calories provided by fat, protein, and carbohydrates in the different variants of KD compared to a typical balanced diet.

Currently, several KD variants are used in clinical practice: the classic ketogenic diet (CKD), the medium-chain triglyceride ketogenic diet (MCTKD), the modified Atkins diet (MAD), and the low glycemic index treatment (LGIT) [1,32]. The CKD is the most restrictive diet and is based on the ketogenic ratio (KR), the ratio between the grams of fat and the sum of the grams of proteins and carbohydrates. Today, CKDs aim to achieve a KR of 4:1 (90% of daily calories derive from fat) or 3:1 (87% of daily calories derive from fat). In CKD fat are represented especially by long-chain triglycerides (LCT). Instead, the carbohydrate allowance is very limited, and the proteins are kept to a minimum to satisfy the requirements for growth. In the MCTKD, 30–60% of the energy is derived from medium-chain triglyceride (MCT) oil, which yields more ketone per kilocalorie of energy than LCT, allowing a decrement of total fat and a higher carbohydrate and protein intake, compared to CKD. MAD is characterized by a KR of 1:1 or 2:1 and a carbohydrate intake of up to 20 g/day. Finally, the LGIT allows a carbohydrate intake of 40–60 g/day limited to food with a glycemic index <50 [1,2]. Figure 1 shows the detailed distribution of macronutrients in the different variants of KD, compared to a balanced diet as recommended by the Italian Society of Human Nutrition [33].

The aim of these variants is to increase the variability, the palatability, and the tolerability of the diet [2], improving the compliance, an important factor for a successful dietary treatment. However, a recent meta-analysis of 11 studies on adult patients reveals a compliance rate of 45% for KDs [34]. Such adherence decreases to 38% when considering only the CKD [34]. This relatively low compliance is related to diet ineffectiveness or diet-intolerability because of severe side-effects or discontinuation caused by psychosocial factors and the restrictiveness of the diet [34].

KDs are not free from side effects and contraindications. The adverse effects most often reported by patients treated with KDs are gastrointestinal problems, increased risk of nephrolithiasis, mineral and vitamin deficiencies, growth retardation, and hyperlipidemia. 

Gastrointestinal side effects include constipation, diarrhea (particularly in patients with MCT supplementation), nausea, vomiting and, rarely, pancreatitis. However, these side effects can improve with continued diet use and following dietary advice, such as increasing fiber and fluid intakes [22]. Pharmacological treatments and diet discontinuations are rarely required [22]. With regard to MCT supplementation, the gradual introduction of MCT should reduce gastrointestinal side effects [22].

Nephrolithiasis is an adverse effect occurring in a 3–7% of children treated with restricted KDs [35,36,37], while no cases of kidney stone have been reported in adults treated with MAD and LGIT [38]. Supplementation with oral potassium citrate seems to help prevent kidney stone formation [35,39].

There are mixed results with regard to an adverse effect of KDs on growth retardation. Some prospective studies reported constant linear growth during the first six months of treatment with CKD [40,41,42], while others have observed a deceleration in height after six months of treatment, in children prescribed with CKD [43,44,45,46,47,48] and MCTKD [49]. Nevertheless, from the available research it is difficult to draw firm conclusions on the effect of the ketogenic diet on growth. Very often the results are confused by the ketogenic dietary management, the follow-up protocol, the duration of treatment, and the pre-existing malnutrition due to the diagnosed disease [50]. Furthermore, the same mechanisms underlying the association between KDs and growth are unclear. One hypothesis involves reduced secretion and bioavailability, during treatment with KDs, of insulin-like growth factor I (IGF-1), one of the major growth effectors in children [47]. Further speculation links the reported growth retardation found in children treated long-term with KDs with reduced levels of serum ghrelin [51]. However, the results concerning the KDs impact on ghrelin concentration are conflicting. Finally, KDs could act differently on growth across the different developmental ages. In a prospective study of 237 children treated with CKD, it was found that older children grew “almost normally”, whereas younger ones had growth retardation [43]. Other studies, on the contrary, did not find any age difference between children who grew as expected and those who had retarded linear growth [44,48]. Prospective studies specifically designed to evaluate the impact of KDs on growth are required before drawing any firm conclusions.

For the lipid profile, the results are discordant. Several prospective studies reported an increment in serum lipids after initiation with CKD, both in children [42,52,53,54] and adults [55,56]. However, in children, these increments are usually transitory, and normalize with diet continuation. Medium-long term studies showed an improvement of the lipid profile after 12 and 24 months [52], with a return to pre-treatment values after this period [57,58]. No study evaluated the long-term effect of CKD on lipid profile in adults. A return to pre-diet values has instead been shown after diet cessation [55]. In adults, early increments in serum lipids have been shown with MAD [38,59,60] and LGIT [38], however, also here, they tend to normalize within a year of treatment [59]. Finally, conflicting results are reported for the impact of MCTKD on blood lipids in children. In a prospective study, an improvement of the lipid profile was observed 4 months after diet initiation [42], however, in another, 7% of the children showed a relevant increase in both total cholesterol and LDL cholesterol values during the MCTKD [61].

Vitamin and mineral deficiencies can also occur because of the limited consumption of fruit and vegetables, grains, and calcium-rich foods. The main deficiencies observed in children treated with restrictive KDs concern vitamins B [1], vitamin C [62], vitamin D and calcium [63], selenium [64,65], and magnesium [66]. Carbohydrates-free multivitamin and mineral products are, therefore, recommended to prevent such deficiencies [1].

Finally, KDs are contraindicated in several specific disorders, particularly those involving fat metabolism. A complete list of disorders in which KDs are contraindicated is reported in the recent international guidelines [1].

In order to increase KDs compliance and reduce the diet’s side effects, thus improving the quality of life and diet of patients treated with KDs, there has, in recent years, been an exponential increment in the availability of special food products usable for KDs.

This review summarizes the food, including sweeteners, and food products for the ketogenic dietary treatment of neurological diseases, describing their nutritional characteristics, giving indications for the use of the different products, and considering their metabolic and health effects. We analyzed the commonly-used KDs foodstuffs with high and low amounts of fat and carbohydrates, and sweeteners, selecting this food from that used by general population. A description is then given of food products specifically designed for KDs, and we illustrate food alternative to traditional cereal-based food.

## 2. Foods for a Ketogenic Diet

### 2.1. Commonly-Used Foods 

Regardless of the variant, KDs mainly include high-fat foods. Table 1 shows the macronutrients content and the KR of the foods common to all KD variants.

In addition to these foods, the less restrictive KD variants allow the use of food products with a low glycemic index. Food products with a low glycemic index available on the Italian and International market are, respectively, reviewed in [68] and [69].

KDs must satisfy protein requirements. However, the maintenance a constant KR limits, especially in CKD, the amount of proteins that can be included in the diet. Generally, in infants and young children, it is still possible to provide 1 g of protein per kg of body weight [70]. This value is, however, below the recommended protein requirement for infants and children aged 1–3 years, but meets the needs of older children [33]. In adults, CKD allows to provide 0.5–0.7 g of protein per kg of body weight in function of the KR. With less restrictive KDs it is easier to satisfy protein requirements. Due to the limit imposed by the KR, proteins must be of high quality (able to satisfy the need for essential amino acids) [70]. For this reason, protein foods are almost exclusively selected from animal foods that are also rich in fat. Particular attention must be paid when such foods need to be cooked before being consumed, as the fats of these products, represented mainly by saturated fatty acids (SFA), tend to be liquefied during cooking and to collect inside the cooking medium. In order to guarantee the correct intake of lipids and to maintain high ketosis, it is important that the lipids released from the food are also consumed. 

With regard to fat intake, what matters to maintain KR is the amount of fat to consume. However, from the nutritional point of view, the dietary fatty acid composition is also important. Several studies have reported an increase in serum lipids and the risk of hyperlipidemia in patients treated with KDs, especially with the more restrictive variants. To reduce the cardiovascular risk, several authors suggest increasing the ratio of polyunsaturated (PUFA) + monounsaturated fatty acids (MUFA) to SFA in the ketogenic meal [70]. A recent prospective study on 12 children with pre-existing hyperlipidemia, and treated for 12 months with KDs (nine CKD and three MCTKD) containing very low SFA (10–11% of daily calorie intake), low dietary cholesterol (less than 80 mg/day), no added trans fatty acids, an optimized intake of MUFA and PUFA, and supplementation of omega-3 and carnitine (if the triglycerides and carnitine levels were, respectively, high and low), showed a significant reduction in total and LDL cholesterol. This suggests that with an optimized dietary fat intake, a healthy serum lipid profile can be achieved [71]. Another prospective study treated 121 children with intractable epilepsy with olive oil-based KDs (the olive oil amount was 80–85% of the total fat) and observed that even the olive oil-based KD caused hyperlipidemia within the first month of treatment. However, this elevated serum level tended to decrease during follow up [72]. In fact, recent international guidelines also suggest the use of olive oil as a possible strategy to prevent hyperlipidemia [1]. It has also been seen that PUFAs induce a greater level of ketosis than SFA [73]. Moreover, PUFAs are natural activators of PPARs, and there is evidence that activation of brain-localized PPARs can influence seizure activity [74]. PUFAs, together with ketone bodies, have been shown to exert neuroprotective activity in neurodegenerative conditions associated with impaired mitochondrial function [4]. Therefore, olive oil, which is particularly rich in MUFA, should be used as the main fat source. To increase the PUFA intake, portions of nuts should be included in the dietary plan. Several epidemiological studies and trials have shown an inverse association between nuts consumption and the risk of hyperlipidemia and cardiovascular disease [75]. However, the effects of nuts in subjects treated with KDs remain unknown. A further strategy to increase PUFA intake consists in the addition of soy lecithin, commercially available in the granular form, to the meal. Finally, vegetable oils rich in PUFA, such as sunflower, wheat germ, and rice oils, can be used in addition to olive oil. In addition to being rich in PUFA, rice oil is also a source of γ-oryzanol, a bioactive compound with antioxidant and hypolipidemic effects [76], and subjects treated with KDs could benefit from its consumption. 

Finally, in order to increase the production of ketone bodies, a portion of the daily lipid intake can also be provided by coconut oil, the main source of MCT. Such addition is possible with all KD variants, not only in MCTKD. For example, if CKD with a KR 3:1 fails to reach therapeutic ketonemia, one valuation can be made of the possibility of replacing a part of the daily lipids with MCTs from vegetable oils, before moving to a more restrictive KD. Indeed, in clinical practice many dietitians adopt a more flexible, patient-tailored approach to KD, combining elements from one or more variants of KD, rather than sticking to a rigid dietary protocol [77].

### 2.2. Sweeteners

As specified in the introduction, in order to be effective, KDs require, according to the variant, the almost total exclusion or the limited introduction of carbohydrates. Hence the need to have foods and ingredients free, or with a limited content, of carbohydrates.

In the food industry, sweeteners are increasingly used as substitutes for sucrose. Indeed, sweeteners, as an alternative to sucrose, allow the sweetening and, at the same time, the reduction of the caloric power and glycemic index of food products. For this reason, such products are widely used in the treatment of obesity and by diabetic subjects. Instead, the use of sweeteners in KDs is a topic that is currently a source of doubt and perplexity, for both health professionals and the families of KDs patients, as the absorption, metabolism and potential effects on ketonemia is relatively unknown. 

Below is a brief description of the most used sweeteners, focusing on their digestion and absorption processes, metabolism, energy value, and their effects on glucose metabolism and the gastrointestinal tract.

#### 2.2.1. Polyols

Polyols, or sugar alcohols, are poorly-digestible carbohydrates commonly obtained by the hydrogenation of sugars. They are used as sugar substitutes to confer a sweet taste to food, and are characterized by a lower degree of sweetness than sucrose, but also have a lower nutritional value. Once ingested, only about one-third of the introduced polyols are absorbed in the small intestine via passive diffusion. The amount absorbed varies depending on the individual polyol [78]. Unabsorbed polyols undergo fermentation by the gut bacteria, resulting in the production of short-chain fatty acids and gases [79]. The low nutritional value of polyols helps consumers reduce energy intake and lose weight. Moreover, the addition of polyols to food determines a lower increment in blood glucose and insulin secretion, which is particularly relevant to diabetic subjects. The major health concern concerning polyols is that such sugar alcohols can have a laxative effect if eaten in large amounts, causing bloating, intestinal gas, and diarrhea. Furthermore, some polyols have known anti-ketogenic effects, which is a concern for subjects treated with KDs (Table 2). 

Table 2 shows the characteristics and properties of the most used polyols.

Erythritol is a small polyol (four-carbon, tetritol) naturally found in fruit and vegetables, mushrooms, and fermented foods like wine and soy sauce. Its sweetness is 60–80% that of sucrose [78]. Being a small molecule, approximately 90% of the erythritol assumed with food is readily absorbed, by diffusion, by the small intestine, while approximately 10% reaches the large intestine. Absorbed erythritol is minimally metabolized by the tissues, and it is essentially excreted unused in the urine [80,81,82]. Therefore, erythritol contributes no calories and does not affect blood glucose levels [83]. Moreover, as erythritol is almost totally absorbed, it causes fewer intestinal symptoms [78,84].

Xylitol is a polyol with the same sweetness as sucrose [78] and it is widely used in various pharmaceutical products, as well as in sugar-free candies and chewing gum. Like other polyols, xylitol is partially absorbed in the small intestine by either passive or facilitated diffusion. Several experts and authorities have proposed that xylitol absorbability is approximately 50% [82]. Once absorbed, xylitol is transferred to the liver where it is dehydrogenated by a non-specific cytoplasmic NAD-dependent dehydrogenase, with consequent production of xylulose, which is first phosphorylated via a specific xylulokinase to xylulose-5-phosphate, an intermediate of the pentose-phosphate pathway and, thus, converted to glucose that is released into the bloodstream or stored as glycogen [85]. It is not known how much glucose is released per gram of xylitol, however, a small (approximately 4 mg/dL) but significant increment of postprandial glycemia has been observed 60 min after the ingestion of a solution containing 25 g of xylitol [86], suggesting a very low glucose production. Unabsorbed xylitol reaches the large intestine where it is fermented by gut bacteria. As a result of its metabolism and fermentation, xylitol contributes 2.4 kcal/g. Moreover, in animal and human studies, xylitol has been reported to have an antiketogenic effect, decreasing ketone body production by the liver and also decreasing blood ketone body levels [87,88,89]. Presumably, this antiketogenic effect is mediated by an indirect mechanism, and is due to a stimulation of insulin release from the pancreas by xylitol [89,90], improving peripheral glucose utilization [79]. With regard to gastrointestinal side effects in healthy humans, these occur after the consumption of an excessive dose of xylitol. In general, single doses ranging from 10–30 g are normally tolerated without diarrhea [91]. In this light, small amounts of xylitol, such as those contained in chewing gum, should not interfere with the ketosis level. However, it is advisable to avoid consuming it in excessive doses.

Mannitol is a polyol with a sweetening potency approximately 50–70% that of sucrose, and it is naturally found in the exudates of certain trees, in marine algae, and mushrooms [78]. Once ingested, approximately 25% of mannitol is absorbed in the small intestine and subsequently excreted not-metabolized in urine [82,92,93]. Unabsorbed mannitol is slowly fermented by gut bacteria with the production of volatile fatty acids [93]. Consequently, mannitol contributes 1.6 kcal/g, but it does not seem to affect blood glucose levels [94]. Concerning the effects of mannitol on the gastrointestinal tract, human studies have shown that a dose of 10–20 g of mannitol have laxative effects [94], while at doses of 40 g, more than of 50% of subjects developed diarrhea [93]. 

Sorbitol, a polyol naturally found in some fruits and vegetables, has a sweetness approximately 50–70% of that of sucrose [78]. The amount absorbed in the small intestine varies depending on the dose ingested. However, overall, an absorption percentage of 25% has been established [82]. This part is fully metabolized in the liver through a non-specific NAD-dependent dehydrogenase, resulting in the production of fructose and then glycogen or glucose, which is then slowly released into the bloodstream [95]. Unabsorbed sorbitol is fermented by gut bacteria to short-chain fatty acids and only small and insignificant amounts of sorbitol are excreted with the feces [95]. As a result of digestion and fermentation, sorbitol contributes 2.6 kcal/g, but it does not seem to affect blood sugar levels [94], even though an increment in the respiratory quotient has been observed [94]. Moreover, in animal studies, sorbitol has been reported to have an anti-ketogenic effect, as sorbitol caused a marked decrement, up to 50%, of the liver production of ACA and BHB [96,97,98]. Additionally, the relation between sorbitol consumption and the appearance of gastrointestinal symptoms seems to be dose dependent [78]. Several human studies have shown that 10 g/day of sorbitol provokes only a mild gastrointestinal problem, like flatulence or bloating, while a dose of 20 g/day can lead to more distressing symptoms, like abdominal pain and diarrhea [99,100].

Maltitol is a disaccharide polyol, consisting of sorbitol and glucose, with a sweetness of 75–90% that of sucrose [78]. It is used in the food industry for the production of hard candies, chewing gum, chocolates, baked goods, and ice cream. Maltitol absorption ranges from 5–80%, however, several authorities have agreed to consider maltitol absorbability as being 45% of the ingested amount [82]. Before its absorption, maltitol needs to be hydrolyzed by the intestinal brush-border disaccharidases in its two components. Thus, the liberated glucose is completely absorbed, whereas the liberated sorbitol is only partially absorbed, as described above. Unabsorbed maltitol passes to the colon where it is fermented by the gut bacteria. As a result of its metabolism and fermentation, maltitol contributes 2.1–2.4 kcal/g. Moreover, in humans, maltitol consumption affects blood glucose level. It has been shown that the oral administration of 50 g of maltitol determined a blood glucose maximal increment of approximately 15–20 mg/dL [101]. An increment of postprandial blood glucose has observed also with lower doses. Only an oral administration of 10 g of mannitol showed no effects on postprandial glycemia [101]. Therefore, caution must be exercised when administering foodstuffs containing maltitol to KD-treated patients. With regard to gastrointestinal distress following maltitol consumption, several studies have shown that doses of 30–35 g of maltitol do not cause significant gastrointestinal symptoms, whereas a dose of 40 g of maltitol caused mild borborygmus and flatus, without laxation. Diarrhea occurred only for very high doses of maltitol (45–90 g) [102,103]. 

Isomalt is a mixture of mannitol and sorbitol and is used in the manufacture of sugar-free hard candies, chewing gum, chocolate, and cough drops. Its sweetness is up to 65% of that attributed to sucrose [78]. After ingestion, isomalt is hydrolyzed by the sucrose-isomaltase and the glucoamylase-maltase complexes of the intestinal brush-border. However, because of its poor affinity with such enzymes, compared to maltose and sucrose, only 10% of ingested isomalt is hydrolyzed and absorbed, while 90% passes into the colon where it is fermented to volatile fatty acids and H_2_ gas by the gut bacteria. As a result of the degradation, isomalt supplies 2 kcal/g. Studies investigating the gastrointestinal symptoms following the ingestion of isomalt have shown that 40 g isomalt leads to an increased incidence of several symptoms, including mild laxation. A reduction of isomalt to 30 g increased tolerance, with evidence of only mild borborygms, mild flatulence, colic, and laxation [104]. In any case, the gastrointestinal symptoms resulting from isomalt ingestion tend to decline with habitual consumption [91].

Lactitol is a disaccharide polyol used in the food and pharmaceutical industries. It has a low sweetness, approximately 30–40% that of sucrose [78]. Once ingested, lactitol passes through the small intestine almost completely unbroken because of the very low activity of β-galactosidase in humans [105,106]. Only 2% of ingested lactitol is, indeed, hydrolyzed in galactose and sorbitol [82]. These two are, respectively, completely and partially absorbed and, on reaching the liver, are metabolized and stored as glycogen or released as glucose into the bloodstream [82]. Unabsorbed lactitol is intensively fermented by the gut bacteria, with the production of volatile fatty acids, particularly butyric acid, and H_2_ gas [79,106]. Consequently, lactitol supplies up to 2.4 kcal/g without raising blood glucose levels [107]. Concerning the gastrointestinal distress following the consumption of lactitol, since almost all of the ingested lactitol reaches the large intestine, it can pull water into the gut causing osmotic diarrhea [91]. Moreover, given the intense production of gases, it can cause flatulence. Human studies have shown that the consumption of low doses of lactitol, up to 10 g/day, causes little gastrointestinal distress [108], while higher doses frequently lead to gastrointestinal symptoms, like flatulence, borborygms, colic, motion frequency, and loose stools [102,104,109,110]. 

#### 2.2.2. Artificial Sweeteners

Artificial sweeteners are synthetic sugar substitutes, but they can be derived from natural sources like herbs or sugar itself. Artificial sweeteners are characterized by high-intensity sweetness, many times sweeter than sucrose. This implies that to achieve the same level of sweetness conferred by sucrose only a minimal amount of artificial sweetener is needed. Furthermore, artificial sweeteners have a low or even no nutritional value, and do not affect blood glucose levels [111].

Table 3 shows the characteristics and properties of the most used artificial sweeteners.

Aspartame is used extensively as a tabletop sweetener and in a wide variety of foods and beverages like chewing gum, yogurt, desserts, and nutritional bars. From the chemical point of view, aspartame is a methyl ester of a dipeptide containing two amino acids: L-aspartic acid and L-phenylalanine. Its calorie content per gram is similar to that of sucrose (4 kcal/g), but aspartame’s sweetening intensity is approximately 200 times that of sucrose. As a result, only a small amount of aspartame is needed to achieve sweetness, leading to virtually no calories from aspartame in sweetened products. Indeed, 125 mg of aspartame approximately replace 25 g of sugar [111]. Aspartame digestion is complete and occurs in the gastrointestinal tract by esterases and peptidases, with the production of methanol, aspartic acid, and phenylalanine, which are all absorbed into the bloodstream [112,113]. Methanol enters the portal circulation and is quickly metabolized to formaldehyde by alcohol dehydrogenase. Formaldehyde, in turn, is then oxidized, by formaldehyde dehydrogenase, to formic acid which is excreted in the urine, or is further metabolized to carbon dioxide and excreted through the breath. The two amino acids derived from the digestion of the aspartame follow, instead, the normal metabolic fate expected for these amino acids, including gluconeogenesis [112,113].

Acesulfame potassium (Acesulfame K, ACK) is a non-nutritive sweetener used in foods and beverages. It has a relative sweetness approximately 200 times that of sucrose. Indeed, 125 mg of ACK replaces approximately 25 g of sugar [111]. Once ingested, acesulfame potassium is rapidly and almost completely absorbed into the systemic circulation [114]. However, it remains not-metabolized and is excreted primarily via the kidneys into urine within 24 h of consumption [111,114]. 

Saccharin is used to sweeten products such as drinks, candies, cookies, and medicines. From the chemical point of view, it is an acid converted into a salt using sodium hydroxide or calcium hydroxide. Saccharin is approximately 300–500 times sweeter than sugar. For this reason, 80 mg of saccharin replaces approximately 25 g of sugar [111]. Approximately 85–95% of dietary saccharin is absorbed in the gastrointestinal tract. Absorbed saccharin binds reversibly to plasma proteins and is distributed via the blood to the body organs. However, saccharin is rapidly excreted not-metabolized with the urine, while unabsorbed saccharin is excreted in the feces [111].

Sucralose is a non-nutritive sweetener derived from sucrose. It is intensely sweet, and has a sweetening potency approximately 600 times that of sucrose, so much so that 40 mg of sucralose can replace approximately 25 g of sugar [111]. The ingested sucralose is poorly absorbed in the gastrointestinal tract and is eliminated not-metabolized through feces. Instead the small amount of absorbed sucralose is excreted not-metabolized through the urine [111,115]. Therefore, sucralose contributes no calories and does not affect blood glucose levels.

Steviol glycosides are sweet tasting compounds extracted from *Stevia rebaudiana* leaves. The most abundant steviol glycosides are stevioside and rebaudioside A. From the chemical point of view, steviol glycosides are diterpene compounds formed by steviol molecule in which the hydrogen atom of the carboxyl group has been replaced by a glucose molecule to form an ester, and a hydroxylic hydrogen has been replaced by combinations of glucose and rhamnose to form an acetal. Once ingested, the glycosidic steviols are not hydrolyzed by the enzymes and acid of the upper gastrointestinal tract [116], so much so that they arrive intact in the large intestine, where they are hydrolyzed to steviol by the gut bacteria. The thus-produced steviol is absorbed and transported to the liver and glucuronidated. The steviol glucuronide is excreted mostly in urine [117]. Concerning the glucose removed from the glycosides, there is no evidence that it is absorbed. Therefore, it is plausible that it is quickly utilized by gut microbiota [111,118].

## 3. Food Products for the Ketogenic Diet

In recent years there has been a notable increase in the number of foodstuffs labeled “ketogenic” on the market. However, not all these products have been developed specifically for the ketogenic dietary treatment of neurological diseases. Many are suggested for the dietary treatment of obesity. Two different categories of ketogenic food products can be distinguished: high-fat products and high-protein low-carbohydrate food products. Furthermore, there are the glucomannan-based products developed for the dietary treatment of obesity and for losing weight, and also these are used in the ketogenic dietary treatment of neurological diseases. 

### 3.1. High-Fat Products

This category includes all foodstuffs specifically developed for the ketogenic dietary treatment of neurological diseases. They are high-fat food products with a quite specific KR. This category includes powder and liquid formulas, MCT-rich products, LCT-rich products, baking mixes, and ready-to-eat foods. The characteristics of each of these products are given below.

Powder ketogenic formula: This is the first food product specifically developed for KDs. It is a high-fat powder food product, with a KR of 4:1 or 3:1, available in different flavors, fortified with docosahexaenoic and arachidonic acids, fiber, minerals, and vitamins, primarily intended for infants from six weeks of age. For infants, it is generally dissolved in water, and used as a meal replacement. For older children and adults, it can be used as a meal substitute and in combination with solid foods, to increase the KR of a meal or to supplement it with vitamins and minerals. Several studies have shown that the use of powder ketogenic formula in KDs is effective, safe, and tolerable for infants and children suffering refractory seizures [119,120]. In a recent study, 27 infants and children with drug-refractory epilepsy, aged between 12 months and five years, were treated with a powder ketogenic formula for four months. Of the 27 children, five were lost to follow-up, 22 remaining to the end of the study. After four months, a reduction of the median frequency of seizures per week was observed: 68.2% of patients showed a 50% reduction in seizure frequency per week, 40.9% showed a 50–90% reduction in seizure frequency per week, and 27.3% showed more than 90% reduction in seizure frequency per week. As far as concerns the adverse effects, 27% of the children developed constipation, one developed gastroesophageal reflux, and another developed hypercholesterolemia. No child discontinued the diet because of the complications. Finally, about 60% of children and their parents reported that the diet was palatable, and quite tolerable [119]. Similarly, in another study involving 10 children with drug-refractory epilepsy, aged nine months to 16 years, a powder ketogenic formula was administered for three months together with solid foods. The authors observed that ketosis was achieved in 7–10 days and at the end of the study, 60% of children showed a 50% or more reduction in in seizure frequency per week, while 10% was seizure-free. The formula was accepted and tolerated by 90% of the children [120]. It was also shown that the addition of the powder ketogenic formula to MAD during the initial month improved the efficacy of the dietary treatment of intractable childhood epilepsy. In this study, 30 children with intractable epilepsy were prospectively followed for two months. During the first month of treatment, MAD was used in combination with a daily 400-calorie powder ketogenic formula. At one month, 80% children had >50% of seizure reduction, of which 37% had >90% of seizure reduction. These findings showed that the likelihood of >50% and >90% seizure reduction was higher than that reported by other studies where only MAD was used. No significant loss of efficacy during the second month, after the powder ketogenic formula was discontinued, was observed. Therefore, the addition of a powder ketogenic formula to the MAD during its initial month may be beneficial for the efficacy of dietary treatment [121]. This product is also indicated for enteral KD [120,122].

Liquid ketogenic formulas: In addition to powder formulations, there are also liquid ketogenic formulas. These are, substantially, ready-to-drink ketogenic formulas with a KR of 4:1 and fortified with docosahexaenoic and arachidonic acids, fiber, minerals, and vitamins. Some versions also have L-carnitine added, and a part of the total fats present in these products consists of MCTs. Liquid ketogenic formulas can be used for infants, children, and adults as meal substitutes or a snack, or in combination with solid foods to increase the KR of a meal or its contribution in vitamins and minerals. Such products have been shown to be safe and generally well accepted by children and their parents. Refractory epileptic children treated almost exclusively with liquid ketogenic formula have been shown to achieve a stable ketosis in a mean time period of seven days. [123]. Moreover, being liquid, such products can be also beneficial for children who are tube-fed and for those in an intensive care setting [123]. 

Products rich in medium-chain triglycerides: MCTs are triglycerides with two or three fatty acids having an aliphatic tail of 6–12 carbon atoms, also called medium-chain fatty acids (MCFAs). These include caproic (C6), caprylic (C8), capric (C10), and lauric (C12) acids. A KD containing 60–75% of calories from lipids induce ketosis if it includes a high proportion of MCT, as, unlike LCTs, they do not require the actions of bile or micellar-chylomicron-mediated absorption into the lymphatic system, being instead diffused into the hepatic portal vein and preferentially converted into ketone bodies in the liver [124,125,126]. This allows an increase in the intake of carbohydrates and proteins and increases the palatability of the diet [127], with little clinical difference in BHB levels when compared to a CKD [128]. On the market, there are products consisting of mixtures of MCTs obtained by extraction from palm and coconut oils, the main food sources of MCTs. Such mixtures, primarily available in the oil form, have different percentage amounts of MCTs, and can have added fat-soluble vitamins, like vitamins A, D, and E. Other available forms include margarines and ready-to-use ketogenic formulas and powders consisting almost exclusively of MCTs. These products are not exclusive to MCTKD, but can also be used in other KD variants, for example, to increase the production of ketone bodies.

Products rich in long-chain triglycerides: Such products are mixtures of LCTs; they contain neither protein nor carbohydrates. They are available in liquid state with a neutral taste and can, therefore, be used to adjust the lipid amount in a meal or a recipe. Alternatively, they can be used as a meal replacement.

Ketogenic baking mixes: To be effective, KDs require, according to the variant, the almost total exclusion or the limited introduction of carbohydrates. This excludes, or limits, foods like bread, pasta, and all bakery products in general. However, these days there are readily available on the market ketogenic baking mixes that substitute grain flours and, thus, allow the domestic production of foods alternative to traditional cereal-based foods. These mixes are basically vegetable oils, in particular sunflower or palm oil, absorbed on a cellulose substrate and rendered in powder form, to which protein sources or other lipid sources, like nut flour, can be added. Currently, there are several versions of these products, with a KR ranging from 4.5:1 to 5:1. 

Ready-to-eat ketogenic products: The number of these products on the market is constantly growing, e.g., biscuits, bread, crackers, savory biscuits, focaccia, and desserts. The nutritional composition of these products is very different from each other. However, two categories can be distinguished on the basis of the KR: ready-to-eat products with a medium-low KR (2:1) and ready-to-eat products with a high KR (3:1 or 4:1). Both categories have the benefit of being ready for consumption, improving diet management due to their palatability and tolerability. Indeed, for a family with a child on a KD, the fact of having safe, ready-to-eat products, with a certified nutritional composition, greatly facilitates the management of the child’s diet, as it reduces the time spent preparing meals. Thus, the child has more foods to choose from, so as to increase the dietary variability, and the fact that the foods consumed are similar to those consumed by healthy peers reduces the feeling of diversity, marginalization and isolation.

### 3.2. High-Protein, Low-Carbohydrate Products

This category includes many food alternatives to traditional cereal-based products, like pasta, bread, biscuits, and desserts. However, these foodstuffs are characterized by a high protein and a low carbohydrate content, and are mainly indicated for the treatment of obesity. The KR and, therefore, the yield in ketone bodies, is, thus, very low and, therefore, they find little use within ketogenic dietary plans, especially in CKD, except in small quantities and balanced with an adequate quantity of fat-rich products.

### 3.3. Glucomannan-Based Products

Glucomannan-based products are foodstuffs not directly involved in the production of ketone bodies; however, being glucomannan a non-digestible soluble dietary fiber obtained from *Amorphophallus konjac* plant tubers [129], these products can be included in a ketogenic food plan as alternative foods to traditional cereal-based foods. Konjac flour is used as low calorie substitute in the food industry for the production of foodstuffs such as noodles and spaghetti [129,130]. Being dietary fiber-rich products, such foodstuffs can delay the stomach emptying, increasing satiety and, for this reason they are mainly used as an anti-obesity agent. Moreover, these products, being low-caloric foods, are also used in KDs as alternative products to traditional cereal based foodstuffs. Beyond that, the inclusion of glucomannan-based products in KDs can lead to some benefits in patients’ health status. Some small-sized studies have shown that glucomannan supplementation can reduce, in both children and adults, the risk of constipation, increasing defecation frequency and the stool bulk without increasing the risk of side effects like abdominal cramping, borborygmi, bloating, and flatulence [131,132,133,134,135,136]. In addition, there is some evidence that glucomannan has prebiotic effects, as it increases the proportion of bifidobacteria and lactobacilli, and reduces the proportion of clostridia to total fecal bacteria [135]. Such effects seem to be partially related to the increment of short chain fatty acids and the decreased fecal pH following the consumption of glucomannan [135]. However, a further double-blind, placebo-controlled randomized trial did not find any amelioration of constipation in children treated with glucomannan compared to the children treated with a placebo [137]. Further benefits, resulting from glucomannans consumption, are related to lipid and glucose metabolism. Recent meta-analysis of randomized controlled trials shows that glucomannans lowered total and LDL cholesterol, triglycerides, and fasting blood glucose, whereas the use of glucomannan did not appear to affect HDL cholesterol or either systolic or diastolic blood pressure [138,139]. In the light of this, there are no contraindications to the use of glucomannan-based products in KDs. However, since these are highly-satiating foods, caution should be exercised when including portions in the dietary plan. Excessive portions of these products can lead to early satiety, with consequent non-consumption of the whole meal, resulting in KR variation and a possible effect on the level of circulating ketone bodies.

## 4. Conclusions

The present review summarizes the foods and the food products available on the Italian market for the ketogenic dietary treatment of neurological diseases. However, it cannot be excluded that there are other products on the international market that have not been taken into consideration in this review. We think that this article is helpful for clinicians trying to promote KDs to patients, and researchers that want to implement the diet into their studies. Moreover, all the information described in this review can be used to provide information for patients treated with KDs and for their caregivers. 

Today, thanks to the awareness campaigns carried out by the associations of families with children treated with KDs, such as Charlie Foundation, Matthew’s friends and, in Italy, Associazione Italiana GLUT1, one hundred years after the birth of KD, food companies have started to develop, and make available on the market, several food products intended for this category of patients. This is very important as it has made it possible to improve the quality of life of patients and their families [140]. In fact, the range of products to choose from has increased, improving dietary variability. Indeed, patients now have access to alternative foods that resemble the traditional ones rich in carbohydrates, making patients feel less different from their healthy peers, and giving them new ingredients for the realization of recipes that are otherwise difficult to make. However, the road is still long. More ready-to-eat ketogenic foods are needed. In addition, the use of KDs for prolonged periods, as in GLUT1-DS patients and in patients with metabolic diseases, requires foods with a lipid profile characterized by a greater presence of MUFA and PUFA and lower in SFA; this is in order to reduce the risk of future dyslipidemia and cardiovascular problems. Moreover, given the reduced consumption of fruit and vegetables, ketogenic foods with added antioxidant and bioactive compounds are really necessary. Finally, much work remains to have these products recognized as medical food products by the various health systems, so that their cost does not fall entirely on the patient, allowing all families, regardless of their income, to access these products.

## Figures and Tables

**Table 1:** Macronutrients content and ketogenic ratio of the most common foods used in a ketogenic dietary plan.

Food	CHO (%)	Protein (%)	Lipids (%)	SFA (%)	MUFA (%)	PUFA (%)	KR
**Vegetable oils**							
Olive oil	0.0	0.0	99.9	14.46	72.95	7.52	
Sunflower oil	0.0	Tr	99.9	11.24	33.37	50.22	
Wheat germ oil	0.0	Tr	99.9	18.5	16.70	60.40	
Rice oil	0.0	0.0	100.0	19.70	39.30	35.00	
Coconut oil	0.0	Tr	99.9	86.80	6.25	1.60	
**Nuts**							
Macadamia nut	5.1	8.0	76.0	12.10	59.06	1.51	5.8:1
Nut	5.1	14.3	68.1	5.57	9.54	40.66	3.5:1
Hazelnut	6.1	13.8	64.1	4.16	38.60	5.20	3.2:1
Almond	4.6	22.0	55.3	4.59	39.44	10.85	2.1:1
Pistachio nut	8.1	18.1	56.1	5.61	36.47	10.66	2.1:1
Peanut	8.5	29.0	50.0	7.13	23.05	14.19	1.3:1
**Fruit**							
Green olives in brine	1.0	0.8	15.0	2.10	10.47	1.68	8.3:1
Avocado	1.8	4.4	23.0	2.48	18.33	1.45	3.7:1
Coconut	9.4	3.5	35.0	30.93	2.38	0.61	2.7:1
**Cheeses**							
Mascarpone	0.3	7.6	47.0	27.55	14.36	1.57	5.9:1
Spreadable cheese	Tr	8.6	31.0	18.52	9.87	0.84	3.6:1
Brie	Tr	19.3	26.9	16.92	7.79	0.80	1.4:1
Gorgonzola	1.0	19.1	27.1	13.10	7.10	0.73	1.3:1
Robiola	2.3	20.0	27.7	16.24	8.46	0.92	1.2:1
Cheddar	0.5	25.0	31.0	18.52	8.52	0.88	1.2:1
**Fish**							
Eel	0.7	14.6	19.6	5.27	8.58	4.56	1.3:1
Mackerel	0.5	17.0	11.1	2.61	4.13	2.46	0.6:1
Salmon	1.0	18.4	12.0	2.97	4.60	3.05	0.6:1
Sardines, canned in oil, drained	0.0	22.3	12.1	2.37	6.60	2.39	0.5:1
**Animal fats**							
Pork lard	0.0	Tr	99.0	33.12	37.14	28.77	
Cow butter	1.1	0.8	83.4	48.78	23.72	2.75	
Cream from cow’s milk with 35% lipids	3.4	2.3	35.0	20.37	10.85	0.94	6.1:1
**Processed meat**							
Sausage	0.6	15.4	26.7	9.44	10.44	4.15	1.7:1
Mortadella	1.5	14.7	28.1	9.25	12.80	3.94	1.7:1
Bacon	0.0	15.8	23.6	7.97	9.82	3.42	1.5:1
Wurstel	1.4	13.7	23.3	6.94	10.81	4.43	1.5:1
**Eggs**							
Hen’s egg yolk	Tr	15.8	29.1	9.82	8.29	4.63	1.8:1
Hen’s whole egg	Tr	12.4	8.7	3.17	2.58	1.26	0.7:1
**Miscellaneous**							
Mayonnaise sauce based on sunflower oil	2.2	4.2	70.0	8.82	23.70	33.35	10.9:1
Coconut flour	6.4	5.6	62.0	53.27	4.08	1.05	5.2:1
Tofu	0.7	8.1	4.8	0.07	1.06	2.70	0.5:1

Abbreviation: CHO: carbohydrates, SFA: Saturated fatty acids, MUFA: Monounsaturated fatty acids, PUFA: Polyunsaturated fatty acids, KR: Ketogenic ratio, Tr: Traces; The nutritional composition of the foods reported in Table 1 have been obtained from the Italian food composition database of the European Institute of Oncology [67].

**Table 2:** Characteristics and properties of the most used polyols.

Sugar Alcohol	Absorption, %	Fermentation, %	Urinary Excretion, %	Sweetness Compared with Sucrose, %	Caloric Value, kcal/g	Antiketogenic Effects	Gastrointestinal Symptoms
Erythritol	90	10	90	60–80	0.2	-	Well tolerated in the usually used doses
Xylitol	50	50	0	100	2.4	Low increment of blood glucose Decrement of ketone bodies production	10–30 g are generally well tolerated without diarrhea
Mannitol	25	75	25	50–70	1.6	-	10–20 g have laxative effects. Higher doses can provoke diarrhea
Sorbitol	25	75	0	50–70	2.6	Decrement of the liver production of acetoacetate and β-hydroxybutyrate	10g can provoke bloating and flatulence. 20 g can lead to abdominal pain and diarrhea
Maltitol	45	55	0	75–90	2.1–2.4	Increment of blood glucose also with low doses of maltitol. Sorbitol is released after digestion of maltitol	30–35 g/day are generally well tolerated. 40 g/day can lead to mild borborygmus and flatus. Higher doses can lead to diarrhea
Isomalt	10	90	0	65	2.0	-	Over 40 g/day can lead to several gastrointestinal symptoms, including mild laxation
Lactitol	2	98	0	30–40	2.4	-	Up to 10g/day are generally well tolerated Higher doses can lead to flatulence, borborygmus, colic, motion frequency and loose stools

**Table 3:** Characteristics and properties of the most used artificial sweeteners.

Artificial Sweeteners	Digestion and Absorption	Metabolism	Sweetness Compared with Sucrose, times	Caloric Value, kcal/g
Aspartame	Completely digested in methanol, aspartic acid, and phenylalanine, which are absorbed in the small intestine	Methanol is excreted with the urine or metabolized to CO_2_ and excreted with breath Aspartic acid and phenylalanine are used for protein synthesis and gluconeogenesis	200	4
Acesulfame K	Almost completely absorbed	Excreted, not-metabolized, into the urine	200	0
Saccharin	85–95% is absorbed	Absorbed saccharin is excreted, not-metabolized, into the urine Unabsorbed saccharin is excreted in the feces	300–500	0
Sucralose	Poorly absorbed	Absorbed sucralose is excreted, not-metabolized, into the urine Unabsorbed sucralose is excreted in the feces	600	0
Steviol glycosides	Not hydrolyzed by human enzymes	Steviol glycosides are hydrolyzed by gut bacteria in steviol and glucose Steviol is absorbed, glucuronidated in the liver and excreted in the urine Glucose is used by gut bacteria	200–300	0
